# Optimization for Simultaneous Determination of a Panel of Advanced Glycation End Products as Biomarkers for Metabolic Diseases

**DOI:** 10.1021/acs.jafc.4c11382

**Published:** 2025-03-10

**Authors:** Weixin Wang, Yingdong Zhu, Shengmin Sang

**Affiliations:** Laboratory for Functional Foods and Human Health, Center for Excellence in Post-Harvest Technologies, North Carolina Agricultural and Technical State University, North Carolina Research Campus, 500 Laureate Way, Kannapolis, North Carolina 28081, United States

**Keywords:** advanced glycation end products, quantification, enzymatic hydrolysis, LC-MS/MS, biomarkers

## Abstract

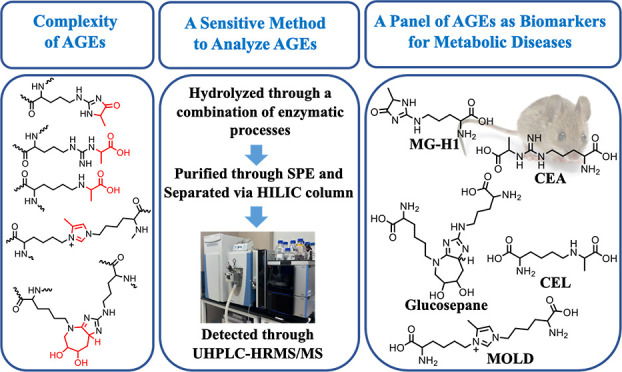

Both dietary and endogenous reactive carbonyl species, such as methylglyoxal (MGO) and glyoxal (GO), react with proteins to generate advanced glycation end products (AGEs), which contribute to metabolic diseases. However, accurately determining individual AGEs in biological samples remains challenging due to the lack of standardized methods. In this study, we optimized and detailed procedures for AGE digestion using enzyme cocktails and separation and detection via high-resolution LC–MS/MS. For the first time, we observed that enzyme backgrounds contained higher levels of methylglyoxal-derived hydroimidazolone 1 (MG-H1) and glucosepane than mouse plasma by 1.4–3 times (e.g., 1512.55 ± 18.89 nM in enzymes vs 496.95 ± 90.91 nM in plasma for MG-H1). Using this optimized method, we quantified fructosyl-lysine and nine AGEs in the plasma, kidneys, and urine of mice. MGO-derived AGEs increased significantly in the plasma and kidneys after MGO treatment. Additionally, both MGO- and GO-derived AGEs were elevated in high-fat-diet (HF)-fed mice compared to low-fat-diet (LF)-fed controls, with further increases in HF-fed mice supplemented with MGO (HFM). This optimized method provides accurate AGE quantification, enabling insights into their role as biomarkers for metabolic syndrome and advancing the understanding of dietary and metabolic contributions to AGE formation.

## Introduction

Nonenzymatic glycation of proteins by reducing sugars or reactive dicarbonyls, such as methylglyoxal (MGO) and glyoxal (GO), forms various advanced glycation end products (AGEs).^1^ Many foods that are associated with type 2 diabetes, such as cookies and carbonated soft drinks, represent exogenous sources of dicarbonyl species.^2−5^ AGE formation leads to protein cross-linking and structural rigidity, which impairs protein function and eventually contributes to severe pathological conditions.^6^ AGEs are particularly relevant in the context of diabetes and age-related diseases, as they accumulate over time, especially in hyperglycemic environments, including cerebrovascular, cardiovascular, and eye-related disorders.^7−9^ Reportedly, MGO-derived AGEs, including methylglyoxal-derived hydroimidazolone 1 (MG-H1), carboxyethyl-arginine (CEA), *N*^ε^-carboxyethyllysine (CEL), and methylglyoxal-lysine dimer (MOLD), are found to be extensively distributed in diabetic and aging tissues.^10^ Besides, the accumulation of sugar-derived AGEs, glucosepane, and 3-deoxyglucosone-derived hydroimidazolone (3DG-H), are also linked to the aging process and cellular dysfunction.^11,12^ Thus, the assessment of major AGEs in the body could be an effective strategy to monitor the progression of diabetes and other age-related diseases.

The determination of AGEs in biological samples is still challenging. ELISA methods are simple and rapid, but this measurement requires specific antibodies for each AGE, and the specificity of the assay is significantly interfered with by the sample matrix, which can result in incorrect estimation of AGE contents.^13,14^ The majority of classic methods of AGE analysis are based on LC–MS techniques, which can provide accurate results.^13,14^ However, protein digestion is required prior to LC–MS analysis.^13,15^ The conventional approach to digest proteins is acid hydrolysis (110 °C for 20–24 h),^13^ but this treatment is not suitable for acid-liable AGEs. More than 90% loss in hydroimidazolone-type AGEs has been observed during the conventional acid hydrolysis.^15^ In addition, the Amadori rearrangement products could be converted into AGEs during acid hydrolysis, leading to an overestimation of AGE concentration in samples.^13,16^ While reductive pretreatment may help mitigate these artifacts,^17^ it also adds complexity to the process. Microwave-assisted acid hydrolysis (MAH) was developed to shorten the time periods for protein hydrolysis and improve the recovery of acid-liable AGEs.^18^ However, the MAH method cannot completely overcome the disadvantages of the conventional approach. Moreover, the lack of reproducibility of microwave-assisted techniques is an apparent drawback for AGE measurements.^19^ Recently, the use of a cocktail of proteolytic enzymes for AGE hydrolysis has been successfully applied in biological samples.^13,20^ However, the procedures for enzymatic hydrolysis are far from being standardized, such as the use of different enzyme combinations from different research groups,^21^ the impact of the background in enzyme systems on the accurate assessment of AGEs in samples, and the impairment of the enzymatic matrix on the sensitivity of the AGE response via LC–MS analysis. Therefore, further optimization of the enzymatic hydrolysis along with LC–MS analysis is warranted.

In this study, we optimized the cocktail of enzymes for AGE hydrolysis and improved the sample cleaning process and sample separation for better LC–MS results. The optimized method was subsequently applied to determine AGE levels in mouse samples collected from MGO and MGO- and high-fat diet (HF)-treatments.

## Materials and Methods

### Chemicals and Materials

*N*^ε^-carboxymethyllysine (CML) (≥95%), CEL (≥98%), and pentosidine were purchased from Cayman Chemical (Ann Arbor, MI). Glyoxal-derived hydroimidazolone 1 (G-H1), glyoxal-derived hydroimidazolone 2 (G-H2), MG-H1, MG-H2, CEA, lactoyl-lysine, furosine, and MOLD were bought from Iris Biotech GmbH (Marktredwitz, Germany). Fructosyl-lysine was purchased from Arctom Chemicals (Newton, MA). Isotope AGEs, G-H1-^13^C_2_, MG-H1-*d*_3_, CML-*d*_4_, CEL-*d*_4_, and furosine-*d*_4_, were bought from Iris Biotech GmbH (Marktredwitz, Germany). *N*^α^-*t*-Boc-lysine (359688, 99%), *N*^α^-*t*-Boc-arginine (408484), 3-deoxyglucosone (75762), perfluoropentanoic acid (PFPA) (396575, 97%), and ammonium formate (70221) were obtained from Sigma-Aldrich (St. Louis, MO). LC/MS grade solvents were purchased from Fisher Chemical (Pittsburgh, PA).

Pepsin (P6887), pronase E (PRON-RO), leucine aminopeptidase (LA) (L6007), LA (L5006), and carboxypeptidase Y (CY) (C3888) were purchased from Sigma-Aldrich (St. Louis, MO). Native porcine prolidase (NATE-0627) and native porcine LA (NATE-1879) were purchased from Creative Enzymes (Shirley, NY). Pierce RIPA buffer was purchased from Thermo Scientific (Waltham, MA).

### Synthesis of 3DG-H

3DG-H was prepared by the incubation of *N*^α^-*t*-Boc-arginine (155 mg, 0.5 mmol) with 3-deoxyglucosone (103 mg, 0.55 mmol) in 2.5 mL sodium phosphate buffer (PBS) (1.0 M, pH 7.4) at 37 °C for 7 days under aseptic conditions following the method in the literature.^15^ The crude product was subjected to reverse-phase (RP) C18 flush chromatography with a Biotage Sfär C18 D -Duo 100 Å 30 μm cartridge (60 g) eluted by H_2_O (200 mL) and 10% MeOH (300 mL).

The Boc-arginine and Boc-3DG-H were coeluted in 10% MeOH. Then the 10% MeOH fraction containing the targeted compound was further purified using semipreparative HPLC, yielding Boc-3DG-H at a retention time of approximately 14.9–15.2 min.

Deprotection of Boc-3DG-H in 0.5 M HCl aqueous solution at room temperature overnight, followed by lyophilization, produced 3DG-H isomers (22.2 mg). ^1^H NMR (600 MHz, CD_3_OD): δ 4.39 (m, 1H, 5-H), 1.99 (m, 1H, 1′-H_A_), 2.22 (m, 1H, 1′-H_B_), 3.59 (m, 1H, 2′-H), 3.50 (m, 1H, 3′-H), 3.66 (m, 1H, 4′-H_A_), 3.75 (m, 1H, 4′-H_B_), 3.39 (m, 2H, 1″-H), 1.79–2.07 (m, 4H, 2″-H and 3″-H), and 4.06 (m, 1H, 4″-H). ^13^C NMR (150 MHz, CD_3_OD): δ 159.4 (2-C), 64.2 (5-C), 33.0 (1′-C), 69.4 (2′-C), 73.4 (3′-C), 67.4 (4′-C), 42.8 (1″-C), 25.5 (2″-C), 29.4 (3″-C), 54.4 (4″-C), and 172.4 (5″-C). HRESIMS *m*/*z*: 319.1599 [M + H]^+^.

### Synthesis of Glucosepane

Glucosepane was prepared by incubating glucose (0.7 g, 3.9 mmol), *N*^α^-*t*-Boc-lysine (3.04 g, 12.3 mmol), and *N*^α^-*t*-Boc-arginine (2.28 g, 8.3 mmol) in 10 mL PBS (1.0 M, pH 7.4) at 70 °C for 17 h, according to the method in the literature.^22^ The crude product was submitted to RP-C18 flush chromatography with a Biotage Sfär C18 D Duo 100 Å 30 μm cartridge (60 g) and eluted with the following gradient: H_2_O (1000 mL), 5% MeOH (1000 mL), 10% MeOH (500 mL), 15% MeOH (2000 mL), and 20% MeOH (1000 mL). The 15% MeOH eluent was combined and dried to get 304.4 mg of crude fraction, including Boc-glucosepane and some Boc-arginine. Then, the crude fraction including Boc-glucosepane was purified by semipreparative HPLC, affording Boc-glucosepane at a retention time of approximately 15.3–15.6 min. Deprotection in 0.5 M HCl aqueous solution at room temperature overnight, followed by lyophilization, gave the desired glucosepane (6.3 mg). ^1^H NMR (600 MHz, CD_3_OD): δ 4.05 (m, 1H, 5-H_A_), 3.57 (m, 1H, 5-H_B_), 3.97 (m, 1H, 6-H), 4.01 (m, 1H, 7-H), 2.13 (m, 1H, 8-H_A_), 2.05 (m, 1H, 8-H_B_), 5.22 (m, 1H, 8-H_a_), 3.84 (m, 1H, 1′-H_A_), 3.38 (m, 1H, 1′-H_B_), 1.80 (m, 2H, 2′-H), 1.58 (m, 2H, 3′-H), 1.88 (m, 2H, 4′-H), 3.62 (m, 1H, 5′-H), 3.37 (m, 2H, 1″-H), 1.80 (m, 2H, 2″-H), 2.00 (m, 2H, 3″-H), and 3.70 (m, 1H, 4″-H). ^13^C NMR (150 MHz, CD_3_OD): δ 169.7 (2-C), 184.5 (3a-C), 52.0 (5-C), 70.7 (6-C), 71.1 (7-C), 33.6 (8-C), 60.0 (8a-C), 54.4 (1′-C), 28.1 (2′-C), 23.9 (3′-C), 32.0 (4′-C), 55.0 (5′-C), 172.6 (6′-C), 43.7 (1″-C), 26.6 (2″-C), 29.6 (3″-C), 54.7 (4″-C), 172.3 (5″-C). HRESIMS *m*/*z*: 429.2449 [M + H]^+^.

### Mouse Studies

#### MGO-Treated Mouse Study

The experimental design and sample collection have been published in our previous study.^23^ Briefly, 6 week-old CD-1 male mice were administered water (control) or 0.12% m/m MGO in water for 6 weeks (*n* = 10). After 6 weeks of treatment, 24 h urine from each group was collected. Subsequently, mice were dissected, and blood, kidney, and liver samples were harvested and stored at −80 °C until analysis. All quality control (QC) materials with low, medium, and high concentration standards were prepared by spiking the corresponding standard mixtures into plasma, kidney, and urine samples from the control group.

#### HF-Fed Mouse Study

The study design and sample collection have been described in our previous studies.^24^ Briefly, 6 week-old C57BL/6J male mice were fed with a low-fat diet (LF) (10% energy from fat), high-fat diet (HF) (45% energy from fat), or HF along with 0.12–0.2% m/m MGO in water (HFM) for 18 wk. At the end of treatments, mice were dissected, and blood and kidney samples were harvested and stored at −80 °C for subsequent analysis.

#### Sample Preparation

Kidney and liver samples (50 mg each) in RIPA buffer (500 μL) were homogenized for 45 s (10 cycles) using an Omni Bead Ruptor Homogenizer (Kennesaw, GA), respectively. The suspension was centrifuged at 13,200*g* for 15 min at 4 °C. The supernatant was collected for further protein digestion. The protein contents in the kidney were decided based on the Bradford Protein Concentration Assay.^25^ In brief, 10 μL of a 30-time diluted kidney homogenate or plasma was mixed with 200 μL of a 5-time diluted Bradford reagent. The mixture was incubated at room temperature for 5 min in the dark. The absorbance at 595 nm for each sample was read by using a plate reader. The creatinine contents in urine were determined with the creatinine assay kit (ab65340, Abcam) according to the manufacturer’s protocol.

Plasma samples were used for protein digestion directly. Urine samples were used for UHPLC-QE+/MS analysis without protein digestion. Briefly, a mixture of IS (2 μM for CML-*d*_4_, CEL-*d*_4_, MG-H1-*d*_3_, and Furosine-*d*_4_; and 10 μM for G-H1-^13^C2) and 10 μL of 5 M HCl were spiked into 50 μL urine. The acidified solution was subsequently loaded onto a Gilson GX-274 ASPEC with a Strata-X-C cartridge (33 μm, 30 mg/1 mL) (Phenomenex) prewashed with 1 mL of 0.1 M HCl in MeOH and 1 mL of 0.1 M HCl in H_2_O. The cartridge was washed with 0.5 mL of 0.1 M HCl in H_2_O, 0.5 mL of 0.1 M HCl in MeOH, and 0.5 mL of 5% NH_4_OH in MeOH in sequential order. The elutes from 0.1 M HCl in MeOH and 5% NH_4_OH in MeOH were combined, dried, and redissolved in 50 μL of 50% MeOH with 0.1 M HCl for UHPLC-QE+/MS analysis. To increase the concentration, 500 μL urine was used, following the procedure mentioned above.

### Conventional Acid Hydrolysis

The conventional acid hydrolysis was carried out by following the literature with some modifications.^13,15^ In brief, 100 μL of the liver homogenate in RIPA (from the control group in the MGO-treated mouse study) was mixed with 100 μL of a 12 M HCl solution. The mixture was incubated at 110 °C for 20 h. After cooling down, 400 μL of acetonitrile (ACN) was added to precipitate the remaining proteins. The supernatant was collected and dried by a gentle stream of N_2_. The residue was reconstituted into 100 μL of 2% ACN with 5 mM PFPA and centrifuged. The supernatant or the diluted supernatant (2–20 times) by 2% ACN with 5 mM PFPA was transferred for HPLC-LTQ/MS analysis.

### MAH

The MAH was conducted by following the literature with some modifications.^18^ In brief, 100 μL of the liver homogenate in RIPA (from the control group in the MGO-treated mouse study) was mixed with 100 μL of 12 M HCl solution and 100 μL of H_2_O. The mixture was loaded onto a CEM Discover SP microwave synthesizer (Matthews, NC) and heated at 150 °C for 1 min and then at 165 °C for another 10 min. The hydrolysate was dried by a gentle stream of N_2_ and redissolved into 100 μL of 2% ACN with 5 mM PFPA. After centrifugation, 10 μL of the supernatant was diluted 10 times with 2% ACN with 5 mM PFPA for HPLC-LTQ/MS analysis.

### Protein Digestion by Enzyme Cocktails

#### General Procedure

The protein digestion for AGE quantifications via enzymatic hydrolysis was performed by following the methods in the literature with some modifications.^26−29^ On day 1, 50 μL of the biological matrix (kidney and liver homogenate and plasma) was mixed with 50 μL of 100 mM HCl, 50 μL of 2 mg/mL pepsin solution (6400 U/mL) in 20 mM HCl, and 25 μL of 2 mg/mL thymol solution in 20 mM HCl. The mixtures were then incubated at 37 °C for 24 h. On day 2, to the incubation mediums from day 1, 75 μL of PBS (100 mM, pH 7.4), 25 μL of 260 mM KOH solution, 50 μL of 2 mg/mL pronase E (3.5 U/mL) in water, and 25 μL of penicillin (1000 units/mL)/streptomycin (1 mg/mL) in 10 mM PBS (pH 7.4) were added, respectively. The mixtures were further incubated at 37 °C for another 24 h. On day 3, the incubation mediums from day 2 were mixed with 25 μL of 1 mg/mL LA solution (11.6 U/mL) in 10 mM PBS (pH 7.4) and 25 μL of 1 mg/mL prolidase solution (114 U/mL) in 10 mM PBS (pH 7.4). The mixtures were continued to be incubated at 37 °C for 24 h. On day 4, to the mediums from day 3, 10 μL IS (the mixture of 2 μM for CML-*d*_4_, CEL-*d*_4_, MG-H1-*d*_3_, and Furosine-*d*_4_ and 10 μM for G-H1-^13^C2), 50 μL standard mixtures at seven levels (S1–S7), and 10 μL 5 M HCl, were spiked. The acidified hydrolysates were subsequently loaded onto a Gilson GX-274 ASPEC with a Strata-X-C cartridge (33 μm, 30 mg/1 mL) (Phenomenex) prewashed with 1 mL of 0.1 M HCl in MeOH and 1 mL of 0.1 M HCl in H_2_O. The cartridge was then washed with 0.5 mL of 0.1 M HCl in H_2_O, 0.5 mL of 0.1 M HCl in MeOH, and 0.5 mL of 5% NH_4_OH in MeOH in sequential order. The eluates from 0.1 M HCl in MeOH and 5% NH_4_OH in MeOH were combined, dried, and redissolved in 50 μL of 50% MeOH with 0.1 M HCl. After centrifugation, the supernatant was removed for UHPLC-QE+/MS analysis. To determine AGE levels in the enzyme background, all buffer and enzymes were added to 50 μL of H_2_O instead of 50 μL of the biological matrix.

#### Hydrolytic Capacity of LA

Similar to the general procedure, pepsin on day 1 and pronase E on day 2 stay the same. LA from three different sources on day 3 was compared. Briefly, three enzyme combinations, including LA (L5006) + prolidase (Pro), LA (L6007) + Pro, and LA (NATE-1879) + Pro, were investigated.

#### Optimization for Enzyme Cocktails

Similar to the general procedure, pepsin on day 1 and pronase E on day 2 stay the same. Three different enzyme combinations on day 3 were investigated, including LA (L6007) alone, LA (L6007) + CY (LA + CY), and LA (L6007) + Pro (LA + Pro).

### HPLC–MS Analysis

#### UHPLC-QE+/MS Analysis over the HILIC Column

UHPLC-QE+/MS analysis was carried out on a Thermo Scientific HPLC equipped with a Vanquish autosampler, pump, column compartment, and a Q Exactive Plus Orbitrap MS system (Thermo Scientific). Chromatographic separation was performed by using an Imtakt Intrada amino acid column (50 × 2.0 mm, 2 μm). Mobile phases were composed of 100 mM ammonium formate in water (mobile phase A) and 0.3% formic acid in acetonitrile (mobile phase B). The flow rate was 0.3 mL/min, and the injection volume was 3 μL. The linear gradient elution had the following profile: 80% B from 0 to 1 min; 80–50% B from 1 to 6 min; 50–0% B from 6 to 10 min; 0% B from 10 to 16 min; 0–80% B from 16 to 16.5 min; and holding 80% B from 16.5 to 18 min. The mass spectrometer was operated with a heated electrospray ionization source under positive ion modes. The key parameters were obtained via tuning the instrument with a mixture of G-H1, pentosidine, and glucosepane, and were provided as follows: spray voltage, 3.50 kV; sheath gas flow rate, 40 arbitrary units (Arbs); auxiliary gas flow rate, 5 Arbs; sweep gas flow rate, 2 Arbs; capillary temperature, 350 °C; S-lens RF level, 70.0; and auxiliary gas heater temperature, 350 °C. The scan mode was PRM with an MS^2^ resolution of 17,500 fwhm and the normalized collision energy of 32–40% depending on the analytes. Data acquisition and processing were performed with Xcalibur 4.2 and Xcalibur Qual Browser (Thermo Scientific).

#### HPLC-LTQ/MS Analysis over the RP-C18 Column

HPLC-LTQ/MS analysis was performed with a Thermo Scientific HPLC equipped with a Dionex Ultimate 3000 XRS Open autosampler, degasser, RS Pump, RS column compartment, and LTQ Velos Pro ion trap mass detector incorporated with heated electrospray ionization interfaces (Thermo Electron, San Jose, CA). Chromatographic separation was performed using a Gemini NX-C18 110A (50 × 2 mm inner diameter, 3 μm) (Phenomenex). Mobile phases were composed of 10 mM PFPA in water (mobile phase A) and 10 mM PFPA in acetonitrile (mobile phase B). The flow rate was 0.2 mL/min, and the linear gradient elution had the following profile: 2% B from 0 to 1 min; 2–10% B from 1 to 1.5 min; 10–25% B from 1.5 to 9 min; 25–100% B from 9 to 10 min; 100% B from 10 to 14 min; 100–2% B from 14 to 15 min; and then 2% B from 15 to 19 min. The injection volume was 5 μL. The electrospray ionization interface was operated under the positive ion mode using a nebulizer at approximately 3.6 kV. Nitrogen gas was used as the sheath gas at a flow rate of 29 arb, and the aux gas was used at a flow rate of 16 arb. Optimized parameters, including temperature (300 °C) and voltage of multipole 0 offset (−7.54 V) and multipole 1 offset (−11.41 V) were obtained via tuning the instrument with a mixture of G-H1, pentosidine, and glucosepane. The selected ion monitoring mode was used to obtain tandem MS. For MS/MS analysis, collision-induced dissociation was conducted using an isolation width of 1.5 Da and a normalized collision energy of 30%. The mass range was measured from 100 to 1500. Data acquisition and analysis were performed using an Xcalibur 2.0 (Thermo Electron, San Jose, CA, USA).

### Semipreparative HPLC Analysis

Semipreparative HPLC was carried out on an Agilent 1260 Infinity II HPLC instrument equipped with a model G7112B HPLC Binary Pump, a model G7129A autosampler, and a model G7115A CoulArray detector (Agilent Technologies, Palo Alto, CA). The chromatographic separation was performed using a Synergi 4 μM Hydro-RP 80 Å column (250 mm × 10 mm). Mobile phases were composed of 0.1% TFA in water (mobile phase A) and 0.1% TFA in acetonitrile for Boc-glucosepane as mobile phase B and 0.1% TFA in MeOH for Boc-3DG-H as mobile phase B. The flow rate was 2.0 mL/min, and the linear gradient elution had the following profile for Boc-glucosepane: 2% B from 0 to 1.0 min; 2–30% B from 1.0 to 5.0 min; 30–40% B from 5 to 7 min; 40–40% B from 7.0 to 16.0 min; 40–100% B from 16.0 to 16.5 min; 100% B from 16.5 to 21.0 min; 100–2% B from 21.0 to 21.5 min; and then 2% B from 21.5 to 26 min, and for Boc-3DG-H: 2% B from 0 to 1.0 min; 2–30% B from 1.0 to 5.0 min; 30–40% B from 5 to 7 min; 40–50% B from 7.0 to 7.1 min; 50–50% B from 7.1 to 15.0 min; 50–100% B from 15.0 to 15.5 min; 100% B from 15.5 to 21.0 min; 100–2% B from 21.0 to 21.5 min; and then 2% B from 21.5 to 26.5 min. The injection volume was 50 μL. The peak of Boc-glucosepane at approximately 15.3–15.6 min was collected, and the peak of Boc-3DG-H at around 14.9–15.2 min was collected.

#### Nuclear Magnetic Resonance Analysis

^1^H- and ^13^C NMR spectra were recorded on a Bruker AVANCE 600 MHz spectrometer (Bruker, Silberstreifen, Rheinstetten, Germany). All compounds were analyzed in MeOD-*d*4. The ^13^C NMR spectra are proton-decoupled.

### Statistical Analysis

The results are expressed as the mean ± standard deviation (SD) for the bar graph. Statistical difference was assessed with two-tailed nonpaired *t* tests and one-way ANOVA with Tukey’s multiple comparisons test using GraphPad Prism 9.4.1. P values indicated on figures follow *p ≤ 0.05; **p ≤ 0.01; ***p ≤ 0.001; ****p ≤ 0.0001.

## Results and Discussion

### Optimization for AGE Detection

**Figure 1:**
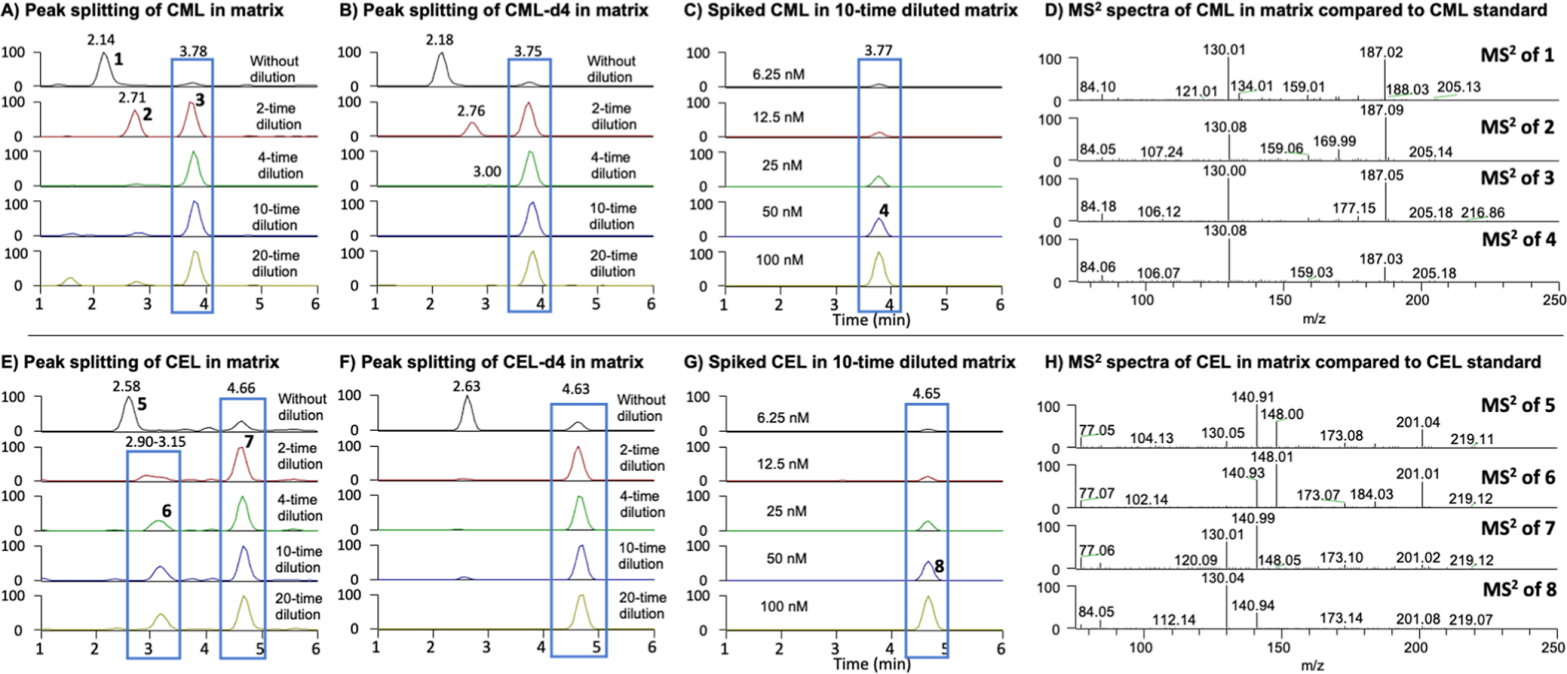
Matrix impacts on CML and CEL profiles. Peak retention time shifting and splitting were observed for CML (A), CML-d4 (B), CEL (E), and CEL-d4 (F) in mouse liver samples at varying dilution levels. Reproducible peaks for CML (C) and CEL (G) were achieved after spiking into 10-fold diluted liver samples. MS^2^ spectra of CML (D) and CEL (H) in the liver matrix were compared to their standards, obtained using a positive LTQ/ESI/MS interface. 10 μL of the liver homogenate in RIPA (from the control group in the MGO-treated mouse study) was hydrolyzed by the enzyme hydrolysis method (the final hydrolysate volume is 100 μL), and then they were diluted 0–20 times in 2% ACN with 5 mM PFPA for LTQ analysis. Then, the STD of 11 AGEs with final concentrations at 6.25, 12.5, 25, 50, and 100 nM were spiked in the 10-times diluted hydrolysate for LTQ analysis.

Digested AGEs are highly polar molecules and are required to be able to be retained to the column for separation. To increase their retention times, ion-pairing agents, such as PFPA, were usually added to mobile phases during LC–MS analysis.^30^ In this study, HPLC-LTQ/MS analysis over a Gemini C18 column with mobile phases containing 10 mM PFPA was tested for analyzing major AGEs, such as CML, CEL, MG-H1, G-H1, and MOLD, in mouse livers collected from the control group in the MGO-treated mouse study after conventional acid hydrolysis. As a result, most of the AGEs generally showed acceptable LC chromatograms (data not shown), except for two important AGEs, CML, and CEL. We noticed that RTs of CML and CEL were drifting and peaks were subjected to splitting, depending on the concentrations of the liver matrix (Figure 1). The CML exhibited two peaks at 2.14 and 3.70 min in the prepared liver sample (100 μL in 2% ACN with 5 mM PFPA, derived from 100 μL liver homogenate in RIPA) without dilution (Figure 1A), aligning with the peaks of CML-*d*_4_ (Figure 1B). When the sample was diluted 2-fold, the CML peak at 2.14 min shifted to 2.71 min, and the intensity of the peak at 3.78 min increased. Further dilution by 4-fold caused the peak at 2.71 min to disappear, but a small peak at 3.00 min still exists in both CML and CML-*d*_4_ samples. After a 10-fold dilution, the peak disappeared in both samples (Figure 1B). These findings suggest that the sample matrix impairs CML detection. This observation was further confirmed by spiking different concentrations of CML (6.25 to 100 nM) into a 10-fold diluted liver matrix (Figure 1C). Additionally, this is confirmed by the observation that MS^2^ spectra of peaks at 2.14, 2.71, and 3.78 min are similar to the one of the authentic CML in a 10-time diluted matrix (Figure 1D). However, the shift and splitting phenomenon for CEL was slightly different from those for CML. CEL exhibited two peaks at 2.58 and 4.66 min in the prepared liver sample without dilution (Figure 1E), aligning with the peaks of CEL-*d*_4_ (Figure 1F). When the sample was diluted 2-fold, there was only one peak in the CEL-*d*_4_ sample but two peaks in the CEL sample. The peak at 3.15 min still exists after further dilution of the sample. This phenomenon was not observed in the 10-fold diluted liver matrix spiked with different concentrations of CEL (6.25 nM to 100 nM) (Figure 1G). Further tandem mass analysis of the peaks at 2.58, 3.15, and 4.66 min shows the major fragment ion of the authentic CEL, *m*/*z* 130.04, could be detected in the peaks at 2.58 and 4.66 min but not in the peak at 3.15 min (Figure 1H), suggesting that the peak at 3.15 min is most likely not CEL.

**Figure 2:**
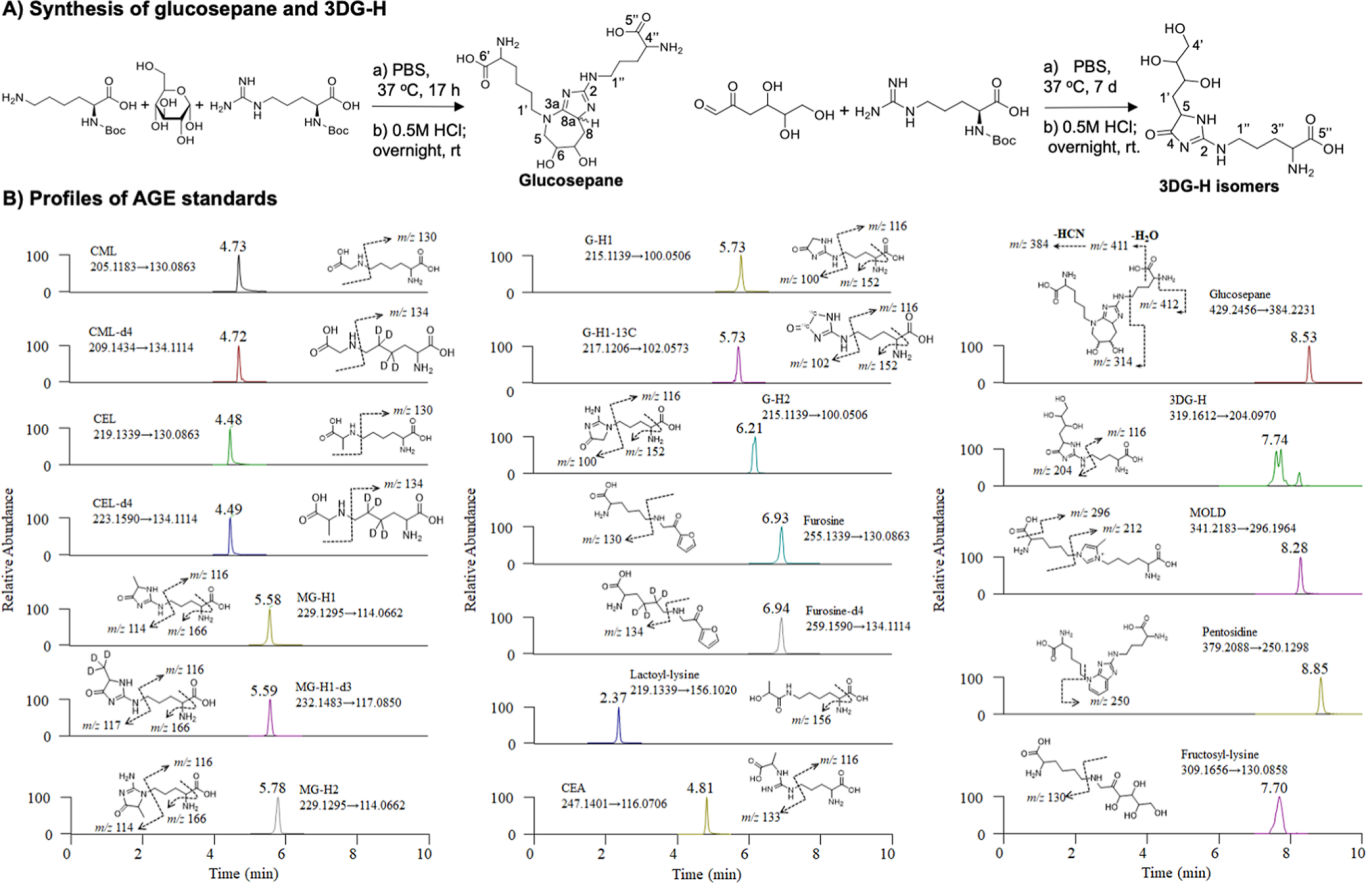
Simultaneous detection of 14 AGEs. (A) Synthesis of glucosepane and 3DG-H. (B) MS^2^ chromatograms and major MS^2^ fragmentations of 14 AGE standards along with 5 isotope AGEs in 50% MeOH obtained by a positive QE+/ESI/MS interface over an Intrada HILIC column. 3DG-H is a mixture of isomers and has multiple peaks.

Although the peak splitting phenomenon for CML and CEL could be minimized in diluted samples, other low levels of AGEs in samples, such as G-H1, became difficult for detection or quantification after a 10-time dilution (data not shown). In addition, the strong acidity (pH ∼ 1.5) of mobile phases containing 10 mM PFPA is on the edge of working pH ranges (1–12) of the Gemini C18 column, leading to rapid deterioration of the reversed-phase column.^30^ In light of these disadvantages from HPLC-LTQ/MS analysis over a Gemini C18 column, the Intrada HILIC column was considered for AGE analysis. According to the literature, the Intrada HILIC column is able to retain polar compounds without ion-pairing regents.^31^ Thus, UHPLC-QE+/MS analysis over an Imtakt Intrada Amino Acid column without ion-pairing agents in mobile phases was utilized to analyze AGE standards in MeOH. It turns out that the Intrada HILIC column is the key pick for successful AGE analysis, allowing us to simultaneously analyze all 13 AGE standards and one Amadori compound standard, including CML, CEL, pentosidine, G-H1, G-H2, MG-H1, MH-H2, CEA, lactoyl-lysine, furosine, MOLD, fructosyl-lysine, glucospane, and 3DG-H, in MeOH with reproducible peak shapes and retention times. Among them, glucospane and 3DG-H were synthesized in-house and characterized by comparing their respective NMR data and MS fragments with the literature (Figure 2A).^15,22^ The major ion chromatograms and MS^2^ fragmentations of all 14 standards are shown in Figure 2B. SPE cleanup steps have been shown to be able to reduce the sample matrix by effectively removing impurities and enriching the target compounds in biological samples.^31^ Therefore, urine samples from the control group in the MGO-treated mouse study were then tested after passing through a Gilson GX-274 ASPEC with a Strata-X-C cartridge, followed by 10-time condensation. As a result, 11 AGEs and fructosyl-lysine could be detected from mouse urine by comparing with corresponding standards (Figure S1). Of which, fructosyl-lysine and 9 urinary AGEs, including CML, CEL, MG-H1, G-H1, CEA, lactoyl-lysine, MOLD, glucospane, and 3DG-H, were major and could be unambiguously identified (Figure S1). In contrast, furosine and pentosidine were minor AGEs in urine (Figure S1) and were barely detectable in mouse tissues as well (Data not shown). Furosine is an amino acid derivative formed during the acid hydrolysis of Amadori compounds.^32^ Therefore, it is reasonable that furosine was barely detectable in mouse tissues when using an enzymatic hydrolysis method.

Thus, UHPLC-QE+/MS analysis over an Imtakt Intrada Amino Acid column along with the Strata-X-C cartridge cleanup steps was optimized for the analysis of AGEs in biological samples.

### Optimization for Enzymatic Hydrolysis

Enzymatic hydrolysis is a more reliable and accurate method for AGEs detection compared with HCl hydrolysis, especially for preserving labile AGEs and minimizing artifacts. While HCl hydrolysis is cost-effective in breaking down proteins for AGE analysis, its acidic conditions can promote artificial AGE formation and degrade labile AGEs, leading to inaccuracies. Although isotope-labeled standards and reductive pretreatment^17^ can mitigate some issues, they add complexity and cannot recover unstable AGEs lost during HCl hydrolysis. Enzymatic hydrolysis, on the other hand, uses specific enzymes under milder conditions, reducing nonspecific degradation and preserving labile AGEs. Any limitations in protein breakdown can be addressed by optimizing enzyme combinations, making enzymatic hydrolysis a superior choice when the accuracy and preservation of labile AGEs are essential.

Protein digestion by the conventional acid hydrolysis broke down acid-liable AGEs, such as MG-H1 and 3DG-H.^15^ We found that the recovery of MG-H1 at 100 nM in the liver matrix dropped to 13.62% after this process (Table S1). While MAH improved MG-H1 recovery to 78.55% at 100 nM in the liver matrix, it still led to a substantial loss of other unstable AGEs, with 3DG-H showing only 12.72% recovery and complete decomposition of glucospane after MAH (Table S1). Considering the reported benefits of enzymatic hydrolysis in overcoming these drawbacks,^13,20^ we evaluated a cocktail of proteolytic enzymes, including pepsin, pronase E, LA, and prolidase,^26−29^ for hydrolyzing AGEs in mouse samples. Pepsin, secreted by the stomach, breaks down proteins into peptides or smaller amino acid groupings. Pronase E, one of the essential enzymes in the procedure, converts both denatured and native proteins into small oligopeptides and individual amino acids. LA cleaves amino acids from the N-terminus of peptides or proteins. In this study, we first tested the influence of pronase E dosages (2, 4, and 20 mg/mL) on AGE hydrolysis from mouse kidney homogenate (50 μL), and unexpectedly observed a nearly proportionate increase of certain AGEs in samples (data not shown), suggesting the presence of remarkable levels of AGEs in enzymes. Enzyme-derived background AGEs can be challenging to fully avoid or resolve, as enzymes inherently contain lysine and arginine residues, which can undergo modification by compounds such as MGO and glucose under certain conditions. One strategy to minimize or normalize enzyme-derived background levels is to use blank digestion controls, where no sample is added, to measure and subtract any background contributions from the enzyme cocktail.

**Figure 3:**
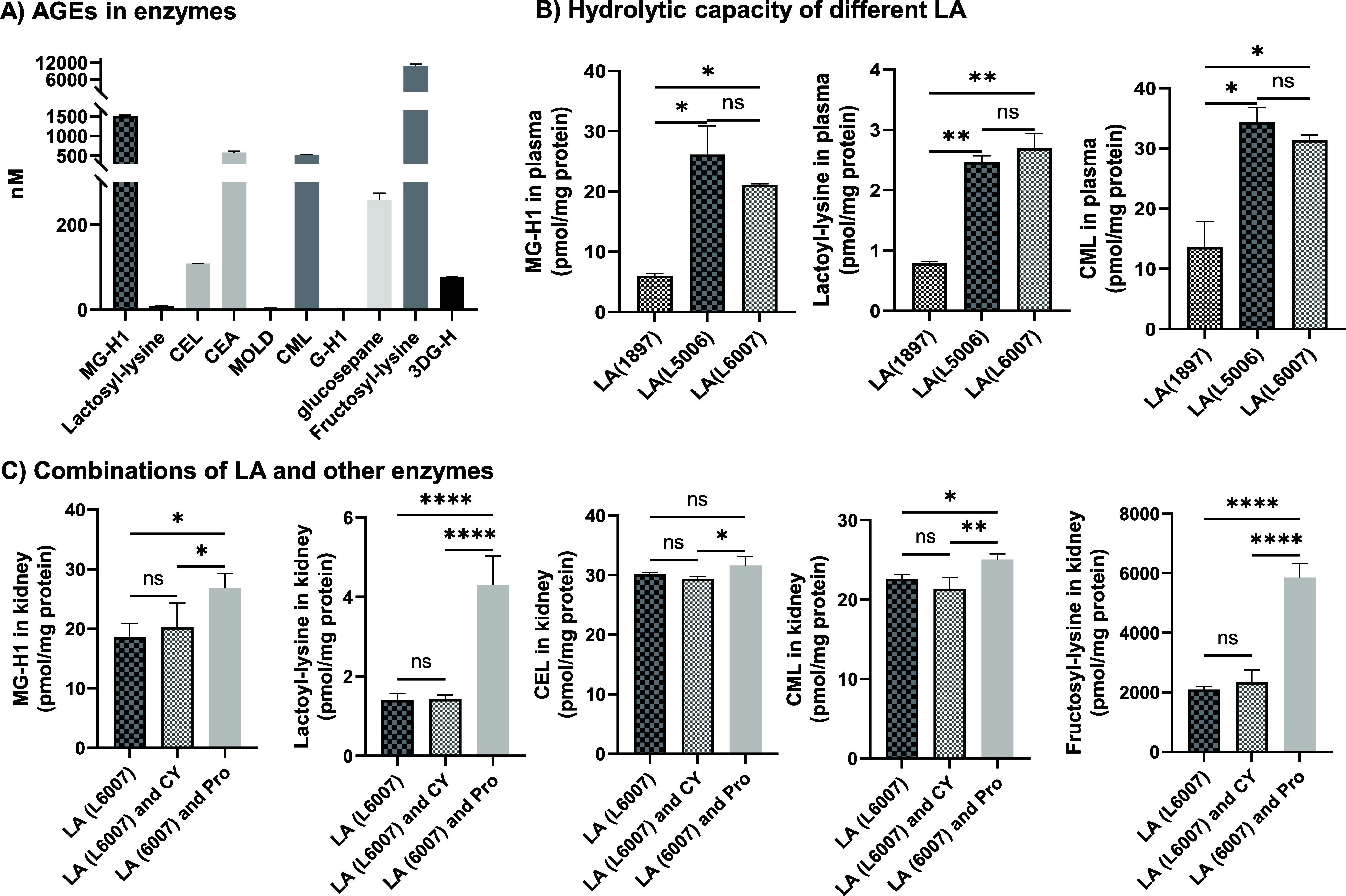
Optimization for enzymatic hydrolysis. (A) Extensive presence of AGEs in enzyme cocktails; (B) hydrolytic capacity of LA from different sources; and (C) optimization for combinations of LA with other enzymes. AGE levels in the enzyme background, plasma, and kidney were determined by following the general procedure for enzymatic hydrolysis of water, plasma, and kidney homogenates, respectively, via a positive QE+/ESI/MS interface over an Intrada HILIC column. Mouse plasma and kidneys were collected from the control mice in the MGO-treated mouse study. Data were expressed as mean ± SD. The *p* values were determined by one-way ANOVA with Tukey’s multiple comparisons test using GraphPad Prism 9.4.1. *p ≤ 0.05; **p ≤ 0.01; ***p ≤ 0.001; and ****p ≤ 0.0001.

Following the general procedure for enzymatic hydrolysis, the blank and mouse plasma and kidney samples (from the control group in the MGO-treated mouse study) were prepared. Prior to the UHPLC-QE+/MS analysis, the method for the instrument was validated. The method validation parameters for major AGEs in urine, plasma, and kidney matrix are provided in Tables S2–S5. Although some RSD values for concentrations below medium QC levels of spiked CEL and CML in the urinary matrix (Table S3), spiked MG-H1, CEL, CML, CEA, and fructosyl-lysine in the kidney matrix (Table S4), and spiked MG-H1, CML, glucospane, and fructosyl-lysine in the plasma matrix (Table S5), fell outside acceptable ranges, the quantitative levels of these AGEs in corresponding mouse samples were primarily within high QC levels in this study. UHPLC-QE+/MS analysis revealed that fructosyl-lysine and 9 AGEs could be detected from the enzymatic background. Among them, MG-H1, CEA, CML, glucosepane, and fructosyl-lysine were found to be present in enzymes in exceptionally high levels (Figure 3A). Particularly, the levels of MG-H1 (1512.55 ± 18.89 nM) and glucosepane (257.51 ± 16.63 nM) in enzymes (Figure 3A and Table S6) outweighed those in mouse plasma by 1.4–3 times (496.95 ± 90.91 nM for MG-H1 and 175.63 ± 12.21 nM for glucosepane) (Table S6). The content of CEA (583.03 ± 30.85 nM) and CML (512.81 ± 10.44 nM) in the enzymatic background (Figure 3A and Table S6) was comparable to mouse plasma (652.53 ± 46.92 nM for CML and 540.77 ± 123.13 nM for CEA) (Table S6). Obviously, the failure to subtract these enzyme-derived AGEs can result in an overestimation of AGE levels in biological samples.

The research on commercial sources resulted in three major availabilities of LA, including L5006, L6007, and NATE-1879. However, the application of LA in the literature was barely specified in catalogue numbers.^26−29^ It is necessary to determine the reactivity of different LAs and standardize the use of a specific LA for the enzymatic hydrolysis. Following the general procedure, plasma and kidney samples from the control group in the MGO-treated mouse study were treated with three different LAs on day 3. We observed that major AGEs in both plasma and kidneys, including MG-H1, lactoyl-lysine, CML, 3DG-H, and fructosyl-lysine, were significantly elevated by L5006 and L6007, compared to NATE-1879 (*p* ≤ 0.05) (Figures 3B, S2, and S3). However, the release of these AGEs in biological samples was not significantly different between L5006 and L6007 (Figures 3B, S2, and S3). These findings demonstrate that aminopeptidases L5006 and L6007 have higher hydrolytic capacities than NATE-1879 does.

To date, research groups utilize different combinations of LA and other enzymes for the hydrolysis on day 3, such as LA + CY,^27^ LA + prolidase (Pro),^15,29,33,34^ and LA alone.^34^ In order to standardize the procedure, the hydrolytic power of these three combinations (LA6007 + CY, LA6007 + Pro, and LA alone) over the control kidney samples was compared. A total of nine AGEs in kidneys after treatment with these three conditions were determined (Figures 3C and S4). We found that fructosyl-lysine and four major kidney AGEs, including MG-H1, lactoyl-lysine, CEL, and CML, were significantly increased after the treatment of LA6007 + Pro, compared to either LA6007 + CY or LA6007 alone (*p* ≤ 0.05) (Figures 3C and S4).

As a consequences, enzyme cocktails consisting of pepsin on day 1, pronase E on day 2, and LA6007 + prolidase on day 3 were standardized for AGE hydrolysis in biological samples. After being cleaned up by a Strata-X-C cartridge followed by condensation, the samples will be submitted to UHPLC-QE+/MS analysis over an Imtakt Intrada Amino Acid column.

### Determination of AGEs in MGO-Treated Mice Using the Optimized Method

**Figure 4:**
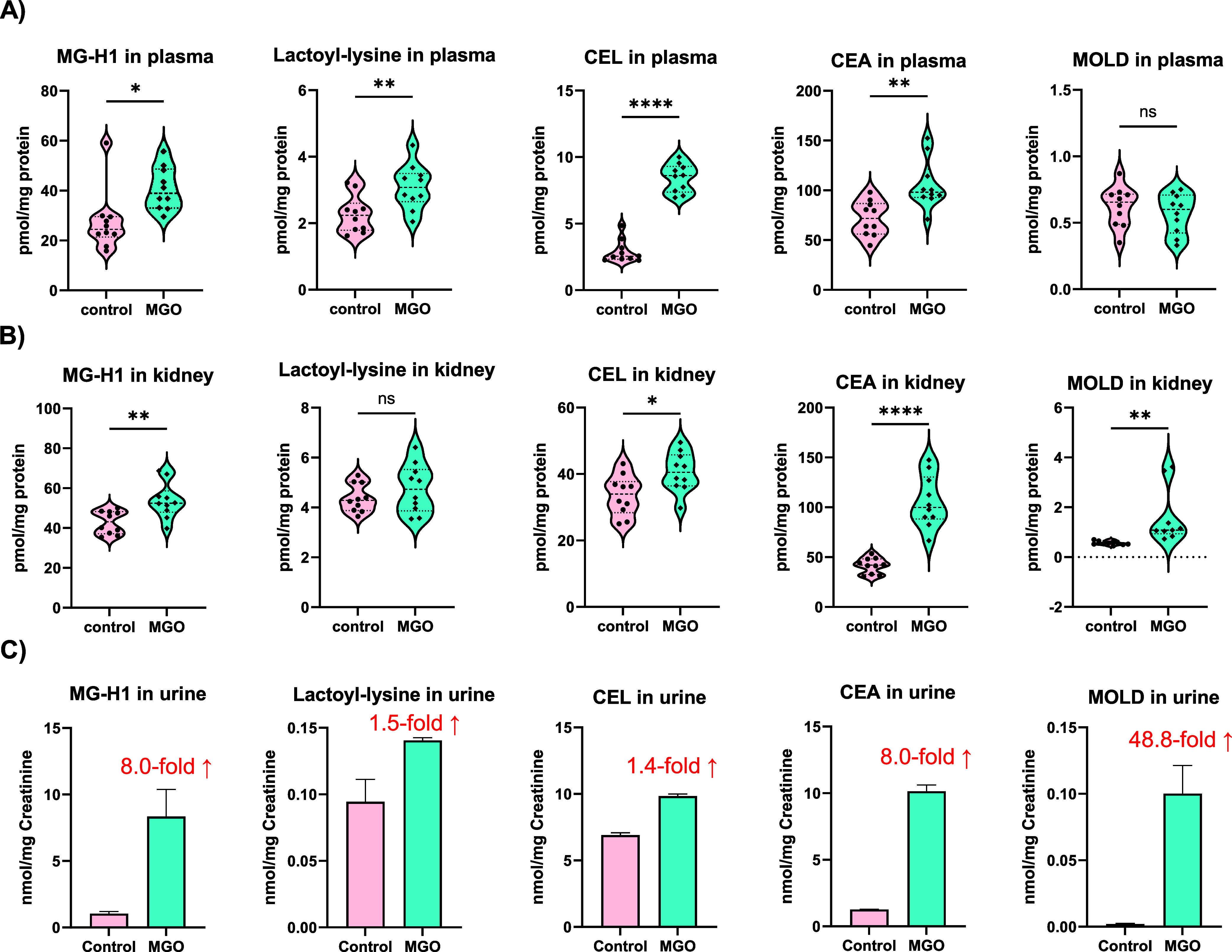
Determination of MGO-derived AGEs in MGO-treated mice by the optimized method. Levels of five MGO-derived AGEs in mouse plasma (A), kidneys (B), and urine (C) after MGO administration were quantified by a positive QE+/ESI/MS interface over an Intrada HILIC column. CD-1 male mice were administered water or 0.12% MGO in water. Plasma, kidney, and 24 h urine were collected after 6 weeks of treatment. Statistical significance (*p* ≤ 0.05) was assessed with two-tailed nonpaired *t*-tests using GraphPad Prism 9.4.1.

The optimized method for AGE analysis was tested in a MGO-treated mouse study. In this study, mice were fed either a regular diet and water (control) or a regular diet and 0.12% MGO in water. Our previous findings revealed that total AGEs in mouse tissues were significantly elevated in MGO-treated group compared to the control.^23^ In the present study, the plasma, urine, and kidneys of mice were prepared and analyzed following the standardized method, and fructosyl-lysine and nine major digested AGEs in mice were qualified and then quantified (Figures 4 and S5). However, argpyrimidine was not detected in our samples, and pentosidine was detectable only in the kidney and only after a 4-fold concentration (data not shown). We found that four out five MGO-derived AGEs in plasma, namely, MG-H1, lactoyl-lysine, CEL, and CEA, were significantly elevated after MGO administration when compared to the control (*p* ≤ 0.05) (Figure 4A). In contrast, fructosyl-lysine and other four non-MGO-related AGEs in plasma, including CML, G-H1, glucosepane, and 3DG-H, showed no significant changes between the treated and control mice (Figure S5A). Meanwhile, four out five MGO-derived AGEs, including MG-H1, CEL, CEA, and MOLD, were significantly higher in the kidneys of MGO-treated mice, compared to the corresponding control (*p* ≤ 0.05) (Figure 4B). In addition, increases in all five MGO-derived AGEs, MG-H1, lactoyl-lysine, CEL, CEA, and MOLD, by 1.4–48.8 times could also be seen in urine after MGO treatment (Figure 4C). Nevertheless, non-MGO-related AGEs in the kidneys and urine of mice leaned toward decrease after MGO treatment (Figure S5B,C). Increases in MGO-derived AGEs in MGO-treated mice were expected when the animal experiment was designed.

Previous studies have reported an elevation of CEL in the kidney following MGO treatment, as determined by ELISA methods.^35^ Furthermore, significant changes in total advanced glycation end-products (AGEs) have been observed in MGO-treated mice, with fluorescence analysis being the primary method of measurement.^23,24,36^ However, due to the diversity of AGEs (fluorescent and nonfluorescent AGEs) in animals, total AGE measurements do not specify which individual AGEs are responsive to the treatments. This study provides optimized techniques to simultaneously determine a panel of individual AGEs in biological samples, allowing for monitoring of the changes of individual AGEs.

### Quantification of AGEs in HF and HF plus MGO-Treated Mice Using the Optimized Method

**Figure 5:**
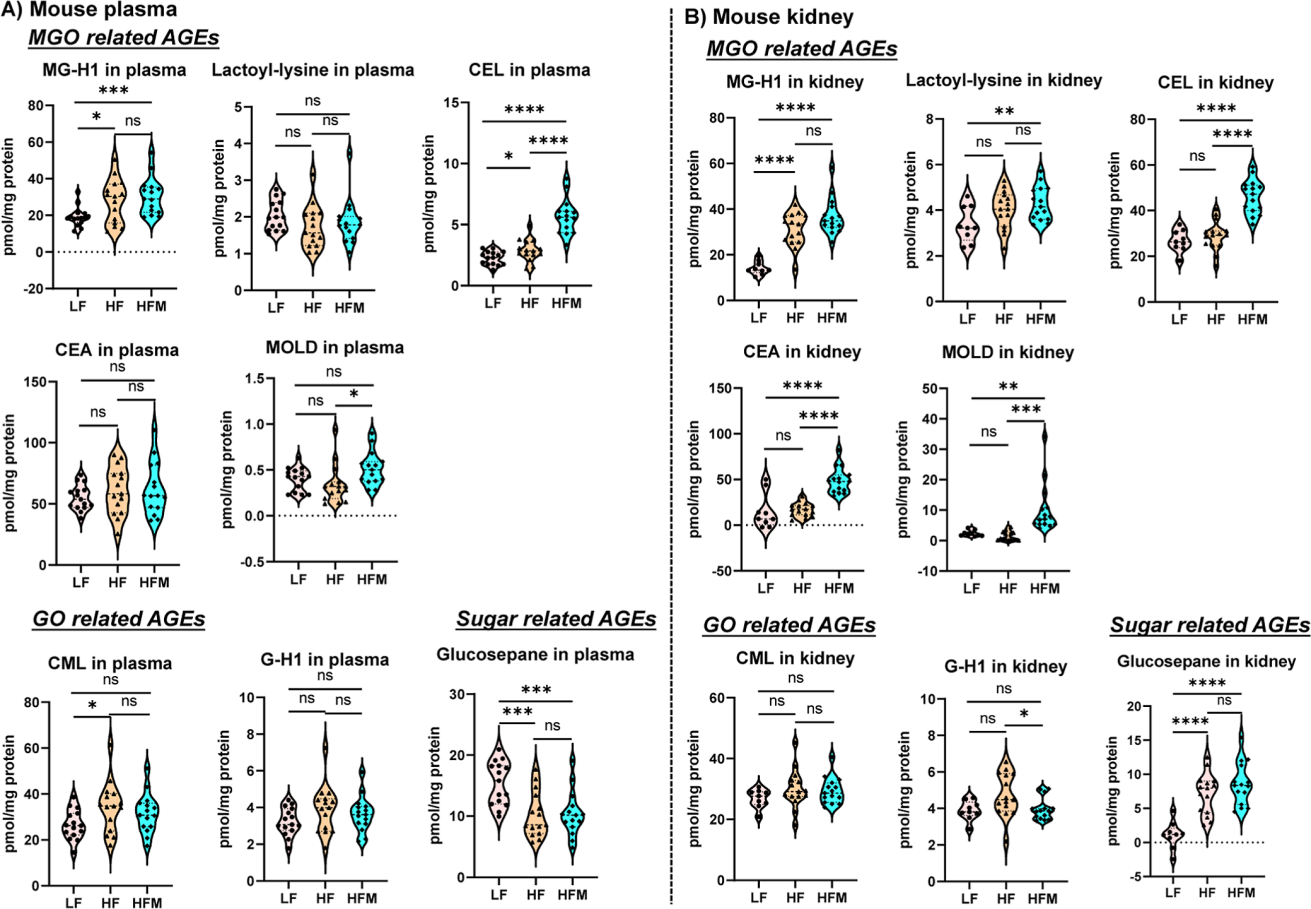
Determination of major AGEs in HF and HF plus MGO (HFM)-treated mice by the optimized method. Levels of eight major AGEs in mouse plasma (A) and kidneys (B) after LF, HF, and HFM administration were quantified by a positive QE+/ESI/MS interface over an Intrada HILIC column. C57BL/6J male mice were fed with a low-fat diet (LF) (10% energy from fat), high-fat diet (HF) (45% energy from fat), or high-fat diet along with up to 0.2% MGO in water (HFM) for 18 wk. At the end of treatments, mice were dissected, and blood and kidney samples were harvested. The *p* values were determined by one-way ANOVA with Tukey’s multiple comparisons test. *p ≤ 0.05; **p ≤ 0.01; ***p ≤ 0.001; and ****p ≤ 0.0001.

The optimized method was further validated in a more complicated mouse model. In this model, three groups of mice, fed a low-fat diet (LF) + water, a high-fat diet (HF) + water, and HF + up to 0.2% MGO in water (HFM), were used. According to our previous findings, HF and HFM treatments significantly increased the plasma and kidney levels of total AGEs.^24^ In the present study, the plasma and kidneys of mice were prepared and analyzed following the standardized method, and ten major digested AGEs in mice were qualified and then quantified (Figures 5 and S6). As a result, HF treatment significantly increased the levels of both MGO- and GO-derived AGEs, MG-H1, CEL, and CML in mouse plasma as well as MG-H1 and glucospane in kidneys, compared to LF treatment (*p* ≤ 0.05) (Figure 5). The combination of HF + MGO (HFM) further enhanced the accumulation of MGO-derived AGEs, such as MG-H1, CEL, and MOLD in plasma and MG-H1, lactoyl-lysine, CEL, CEA, and MOLD in kidneys, compared to the respective LF or HF groups (*p* ≤ 0.05) (Figure 5). Whereas most of GO- and sugar-related AGEs were less sensitive to HFM treatments when compared to HF treatments (Figures 5 and S6). It has been reported that administering an HF diet to mice induces significant increases in MGO and GO levels in tissues and body fluids,^24,37^ suggesting an elevation of MGO- and GO-derived AGEs in the body. Our data revealed significant increases in both MGO- and GO-derived AGEs between HF and LF mice as well as significant increases in MGO-derived AGEs between HFM and HF mice. These findings precisely reflect the effects of different treatments, demonstrating that the current optimized method can provide accurate and reliable results for simultaneously measuring individual AGEs in biological samples.

In the present study, pentosidine and furosine could be detected but not quantified from the tissues and body fluids of mice due to the low levels. Pentosidine has been proposed as a potential biomarker for diabetic cardiovascular risk.^38^ Its levels were significantly affected by glycemic control and renal function.^38^ Reportedly, the urinary concentration of pentosidine in healthy subjects was around 14 nM.^39^ On the other hand, furosine excretion has also been associated with cardiovascular and all-cause mortality.^40^ Given subjects with cardiovascular diseases, these two biomarkers could be substantially increased and reach quantitative levels. The lactylation of lysine residues may have implications for Alzheimer’s disease.^41^ The production of lactoyl-lysine is regulated by the enzymes glyoxalase 1 (GLO1) and glyoxalase 2 (GLO2).^42^ Lactoyl-lysine is formed from the acyl transfer of lactoylglutathione, a secondary glycolytic intermediate produced by GLO1 and broken down by GLO2 into lactate. Previous studies suggest that MGO supplementation increases intracellular levels of MGO and accelerates the formation of MGO-derived AGEs.^43^ This also leads to decreased levels of GLO1 and GLO2 in different animal tissues,^23^ complicating the regulation of lactoyl-lysine levels. Regardless, the optimized method validated in this study has potential to precisely monitor and assess pentosidine and furosine as well as other digested AGEs in patients with diabetes and other age-related diseases.

In conclusion, we enhanced and finalized the detailed protocol for AGE digestion using an enzyme cocktail consisting of pepsin on day 1, pronase E on day 2, and LA6007 + prolidase on day 3. We also optimized AGE separation and detection through UHPLC-QE+/MS analysis over a HILIC column with SPE cleanup. With this optimized method, a panel of AGE standards can be analyzed simultaneously. This method was subsequently applied to quantify ten individual AGEs in mice, including CML, CEL, MG-H1, lactoyl-lysine, CEA, G-H1, glucosepane, MOLD, fructosyl-lysine, and 3DG-H. We observed significant increases in major MGO-derived AGEs in mouse plasma and kidneys, such as MG-H1, lactoyl-lysine, CEL, CEA, and MOLD, following MGO treatment. Additionally, our results showed significant increases in both MGO- and GO-derived AGEs between HF- and LF-fed mice as well as in MGO-derived AGEs between HFM- and HF-fed mice. These changes in individual MGO- and GO-derived AGEs, measured using the optimized method, accurately reflected treatment differences, demonstrating the method’s capability to provide precise and reliable results for the simultaneous measurement of individual AGEs in biological samples.

## Supporting Information Available

Quantification parameters for measuring AGEs in biological samples, concentration of fructosyl-lysine and 9 urinary AGEs in the hydrolyzed enzyme and plasma matrix solution, LC/MS profiles of major AGEs in mouse urine, optimization of enzymatic methods, and determination of AGEs in biological samples (PDF)
