# Supplementary material for: Optimization for Simultaneous Determination of a Panel of Advanced Glycation End Products as Biomarkers for Metabolic Diseases
Source: J Agric Food Chem. 2025 Mar 10;73(11):6970–80. doi: 10.1021/acs.jafc.4c11382 (PMC11926871; doi:10.1021/acs.jafc.4c11382)
Supplement: Supplementary file 1 — jf4c11382_si_001.pdf [file jf4c11382_si_001.pdf]

Supplemental Information

Optimization for Simultaneous Determination of a Panel of Advanced Glycation End Products as Biomarkers for Metabolic Diseases

Weixin Wang, Yingdong Zhu, Shengmin Sang*

Laboratory for Functional Foods and Human Health, Center for Excellence in Post-Harvest Technologies, North Carolina Agricultural and Technical State University, North Carolina Research Campus, 500 Laureate Way, Kannapolis, North Carolina, 28081, USA

***Corresponding Author**

Shengmin Sang, Ph.D.; Email: ssang@ncat.edu or shengminsang@yahoo.com

Table of Contents

Table S1. Recovery of major AEGs after respective conventional acid hydrolysis and microwave-assisted acid hydrolysis (MAH)

Table S2. Linearity for measuring fructosyl-lysine and nine AGEs in mouse urine, kidney and plasma

Table S3. Precision and accuracy for measuring fructosyl-lysine and nine AGEs in mouse urine

Table S4. Precision and accuracy for measuring fructosyl-lysine and nine AGEs in mouse kidney

Table S5. Precision and accuracy for measuring fructosyl-lysine and nine AGEs in mouse plasma

Table S6. Concentration of fructosyl-lysine and nine AGEs in hydrolyzed enzyme and plasma matrix solution (nM)

Figure S1. Twelve AGEs from mouse urine

Figure S2. Hydrolytic capacity of leucine aminopeptidase from different sources for AGEs in plasma samples

Figure S3. Hydrolytic capacity of leucine aminopeptidase from different sources for AGEs in kidney sample

Figure S4. Optimization for enzyme cocktails

Figure S5. Determination of major AGEs in MGO-treated mice by the optimized method

Figure S6. Determination of major AGEs in HF and HF plus MGO-treated mice by the optimized method

Table S1. Recovery of major AEGs after respective conventional acid hydrolysis and microwave-assisted acid hydrolysis (MAH)

AGEs (100 nM)	Recovery after conventional acid hydrolysis (%)	Recovery after MAH (%)
**CML**	101.87	103.15
**CEL**	104.55	101.52
**MG-H1**	13.62	78.55
**MOLD**	104.26	77.96
**Pentosidine**	105.01	103.15
**G-H1**	N.A.	118.16
**Furosine**	N.A.	60.98
**3DG-H**	N.A.	12.72
**Glucosepane**	N.A.	0

The recovery of AGEs were assessed by spiking specific AGE standards (100 nM as the final concentration) into mouse liver homogenates before and after hydrolysis. Conventional acid hydrolysis was performed with 12 M HCl at 110 ^{o}C for 20 h. MAH was conducted with 4 M HCl at 150 °C for 1 min and then 165 °C for another 10 min. All samples were diluted 10 times for HPLC-LTQ/MS analysis. Mouse liver samples were collected from the control mice in MGO-treated mouse study. NA, not applied.

Table S2. Linearity for measuring fructosyl-lysine and nine AGEs in mouse urine, kidney and plasma

	urine			kidney			plasma		
	curve	R^2	Range	curve	R^2	Range	curve	R^2	Range
MG-H1	y = 0.0013x - 0.1141	0.9997	39.06-80000 nM	y = 0.0011x + 0.1011	0.9997	19.53-10000 nM	y = 0.0012x + 0.1628	0.9991	19.53-10000 nM
Lac-lysine	y = 0.0009x - 0.0447	0.9994	9.77-20000 nM	y = 0.0073x - 0.0563	0.9903	4.88-625 nM	y = 0.0034x - 0.0732	0.9872	4.88-1250 nM
CEL	y = 0.0023x + 0.3688	0.9994	9.77-20000 nM	y = 0.0021x + 0.0714	0.9997	4.88-2500 nM	y = 0.0022x + 0.0132	0.9997	4.88-2500 nM
CEA	y = 0.0004x - 0.0898	0.9992	39.06-80000 nM	y = 0.0006x + 0.0999	0.9992	39.06-10000 nM	y = 0.0006x + 0.0621	0.9852	39.06-10000 nM
MOLD	y = 0.0755x + 3.1117	0.9998	9.77-20000 nM	y = 0.0227x + 0.3076	0.9991	4.88-2500 nM	y = 0.0139x - 0.201	0.9988	4.88-2500 nM
CML	y = 0.0025x + 1.314	0.9997	39.06-80000 nM	y = 0.0025x + 0.0999	0.999	19.53-10000 nM	y = 0.0025x + 0.3479	0.9997	156.25-10000 nM
G-H1	y = 0.0008x + 0.0376	0.9999	9.77-20000 nM	y = 0.0007x - 0.0041	1	19.53-2500 nM	y = 0.0007x - 0.0048	1	4.88-2500 nM
glucosepane	y = 0.0005x - 0.0278	0.999	9.77-20000 nM	y = 0.0001x + 0.0011	0.9954	78.13-2500 nM	y = 5E-05x - 0.0003	0.9982	39.06-2500 nM
3DG-H	y = 0.0008x + 0.2432	0.9993	39.06-80000 nM	y = 0.003x - 0.0563	0.992	78.13-2500 nM	y = 0.0007x - 0.0713	0.9982	78.13-5000 nM
Fructosyl-lysine	y = 0.0005x + 12.637	0.9955	312.50-500 μM	y = 0.0006x + 12.963	0.9862	4.88-625 μM	y = 0.0003x - 1.9034	0.9633	39.06-625 μM

The linearity of AGEs were obtained by spiking specific AGE standards at seven levels into mouse urine, kidney, and plasma samples. Mouse plasma and kidney samples were hydrolyzed by enzyme cocktails following the general procedure and then passed through SPE. Mouse urine samples were passed through SPE directly. All samples were analyzed by UHPLC-QE+/MS. Mouse urine, kidney, and plasma samples were collected from the control group in MGO-treated mouse study.

Table S3. Precision and accuracy for measuring fructosyl-lysine and nine AGEs in mouse urine

Analytes in the mouse urine	vLow				Low				Middle				High			
	intra-day		inter-day		intra-day		inter-day		intra-day		inter-day		intra-day		inter-day	
	accuracy (%)	RSD (%)	accuracy (%)	RSD (%)	accuracy (%)	RSD (%)	accuracy (%)	RSD (%)	accuracy (%)	RSD (%)	accuracy (%)	RSD (%)	accuracy (%)	RSD (%)	accuracy (%)	RSD (%)
MG-H1 (250/500/2000/4000 nM)	108.99	22.40	112.55	22.74	102.59	14.04	105.38	16.85	98.03	6.72	98.13	4.40	96.04	3.92	95.81	3.14
Lac-lysine (62.5/125/500/1000 nM)	109.28	6.05	127.00	10.92	93.61	18.01	110.19	15.30	87.89	2.29	96.46	7.31	86.81	1.61	92.41	5.01
CEL (62.5/125/500/1000 nM)	*544.24*	*67.76*	*246.56*	*128.88*	*315.66*	*53.47*	*264.99*	*38.15*	*36.21*	*96.70*	*38.48*	*98.23*	127.68	12.83	120.06	18.65
CEA (250/500/2000/4000 nM)	113.13	15.95	121.33	18.12	113.62	8.72	113.12	10.73	106.97	6.68	106.71	7.78	105.16	13.83	100.44	15.48
MOLD (62.5/125/500/1000 nM)	101.03	10.83	94.65	11.60	93.16	7.55	90.67	8.27	91.32	9.85	90.47	9.69	93.32	10.73	91.80	9.33
CML (250/500/2000/4000 nM)	*290.27*	*124.13*	*173.83*	*143.99*	*261.68*	*32.01*	*258.19*	*36.32*	*34.28*	*61.87*	*49.91*	*40.47*	119.75	3.03	115.47	13.06
G-H1 (62.5/125/500/1000 nM)	122.60	9.42	100.01	29.35	97.50	21.42	99.16	16.67	96.94	6.46	95.71	7.16	97.50	3.29	95.87	4.78
glucosepane (62.5/125/500/1000 nM)	106.65	22.60	124.83	24.22	98.10	16.24	116.02	17.99	96.07	4.82	109.09	10.17	96.08	3.15	105.44	7.64
3DG-H (250/500/2000/4000 nM)	136.22	15.06	152.88	21.48	79.99	29.07	137.53	42.78	101.99	5.28	126.35	16.79	107.84	1.28	121.84	9.71
Fructosyl-lysine (15.625/31.25/125/250 μM)	*-102.00*	*-3326.80*	*-600.48*	*-382.56*	*168.95*	*333.90*	*253.10*	*253.70*	*-335.71*	*-72.89*	*-256.73*	*-116.74*	*63.43*	*412.34*	*101.22*	*242.34*

Precision and accuracy were assessed by analysis of quadruplicate QC samples on the same day (intra-day) or three consecutive validation days (inter-day). Mouse urine samples were passed through SPE directly. Samples were analyzed by UHPLC-QE+/MS. Mouse urine samples were collected from the control group in MGO-treated mouse study.

Table S4. Precision and accuracy for measuring fructosyl-lysine and nine AGEs in mouse kidney

Analytes in mouse kidney	vLow				Low				Middle				High			
	intra-day		inter-day		intra-day		inter-day		intra-day		inter-day		intra-day		inter-day	
	accuracy (%)	RSD (%)	accuracy (%)	RSD (%)	accuracy (%)	RSD (%)	accuracy (%)	RSD (%)	accuracy (%)	RSD (%)	accuracy (%)	RSD (%)	accuracy (%)	RSD (%)	accuracy (%)	RSD (%)
MG-H1 (250/500/2000/4000 nM)	*140.51*	17.61	*141.81*	14.57	115.49	8.98	114.88	6.49	109.19	6.33	110.17	4.23	108.62	2.37	108.05	3.20
Lac-lysine (62.5/125/500/1000 nM)	52.20	7.39	50.72	7.92	111.79	3.02	109.63	4.40	110.84	13.04	107.21	13.73	108.44	2.21	106.31	3.73
CEL (62.5/125/500/1000 nM)	*138.89*	2.38	*149.28*	7.31	*139.56*	9.60	*136.43*	6.91	112.01	2.50	111.71	2.45	107.22	2.86	106.79	3.22
CEA (250/500/2000/4000 nM)	*208.64*	13.01	*198.31*	13.79	89.40	5.37	92.26	4.68	105.16	5.46	108.29	6.31	105.18	14.03	108.09	10.76
MOLD (62.5/125/500/1000 nM)	91.20	9.35	90.45	8.38	99.77	1.59	101.46	2.79	99.46	3.26	103.02	4.11	102.24	5.24	103.35	4.20
CML (250/500/2000/4000 nM)	128.61	13.50	*140.97*	12.97	119.49	9.02	124.14	7.78	107.27	2.26	106.19	3.15	106.60	2.53	105.42	4.25
G-H1 (62.5/125/500/1000 nM)	82.17	24.16	96.05	30.11	81.45	16.21	93.18	20.15	107.68	4.23	102.54	8.16	108.28	4.00	105.45	3.75
glucosepane (62.5/125/500/1000 nM)	94.27	61.13	99.62	102.93	88.50	56.33	122.62	41.34	110.66	20.91	126.89	18.10	122.95	14.64	132.66	14.05
3DG-H (250/500/2000/4000 nM)	104.96	33.27	104.85	37.11	115.17	3.92	121.86	6.96	98.51	6.68	105.48	9.35	100.05	11.97	104.24	11.00
Fructosyl-lysine (15.625/31.25/125/250 μM)	108.11	20.56	107.82	*45.85*	*245.73*	9.01	*253.96*	13.75	*153.90*	5.61	*159.02*	7.67	138.84	7.61	136.42	6.57

Precision and accuracy were assessed by analysis of quadruplicate QC samples on the same day (intra-day) or three consecutive validation days (inter-day). Mouse kidney samples were hydrolyzed by enzyme cocktails following the general procedure and then passed through SPE. Samples were analyzed by UHPLC-QE+/MS. Mouse kidney samples were collected from the control group in MGO-treated mouse study.

Table S5. Precision and accuracy for measuring fructosyl-lysine and nine AGEs in mouse plasma

Analytes in mouse plasma	vLow				Low				Middle				High			
	intra-day		inter-day		intra-day		inter-day		intra-day		inter-day		intra-day		inter-day	
	accuracy (%)	RSD (%)	accuracy (%)	RSD (%)	accuracy (%)	RSD (%)	accuracy (%)	RSD (%)	accuracy (%)	RSD (%)	accuracy (%)	RSD (%)	accuracy (%)	RSD (%)	accuracy (%)	RSD (%)
MG-H1 (250/500/2000/4000 nM)	*145.38*	4.75	*144.57*	8.40	122.56	6.11	121.64	5.12	101.42	1.07	101.72	2.05	102.53	2.28	101.75	1.70
Lac-lysine (62.5/125/500/1000 nM)	84.32	3.15	85.34	7.61	75.43	13.37	74.65	13.60	124.01	5.56	125.39	6.71	67.80	4.86	67.66	4.40
CEL (62.5/125/500/1000 nM)	116.68	5.06	113.66	10.20	102.40	2.88	102.76	1.96	100.36	1.38	100.69	1.18	99.63	2.64	100.12	1.60
CEA (250/500/2000/4000 nM)	101.85	29.33	118.99	23.09	124.58	*51.46*	130.62	*51.91*	69.34	15.64	71.27	12.65	101.12	16.17	102.15	13.53
MOLD (62.5/125/500/1000 nM)	106.47	17.71	110.86	16.94	102.63	17.44	102.69	15.97	97.95	13.93	99.86	11.19	87.00	9.80	87.98	9.27
CML (250/500/2000/4000 nM)	*142.43*	9.89	*146.85*	9.34	115.46	5.09	115.50	3.95	106.40	1.90	105.80	2.64	103.83	1.81	103.22	2.20
G-H1 (62.5/125/500/1000 nM)	105.58	10.38	85.92	22.56	100.62	10.95	100.13	11.66	105.03	3.37	107.26	3.79	105.70	2.26	106.92	1.72
glucosepane (62.5/125/500/1000 nM)	78.93	*74.96*	*136.95*	*69.94*	86.53	25.60	107.03	38.92	131.30	25.07	128.44	24.23	82.47	23.93	96.10	16.05
3DG-H (250/500/2000/4000 nM)	60.71	35.63	95.65	45.11	56.01	10.48	63.02	18.03	106.92	11.54	115.50	12.25	81.59	3.78	87.27	6.05
Fructosyl-lysine (15.625/31.25/125/250 μM)	*35.95*	*898.38*	*93.10*	*324.12*	*67.43*	*61.56*	76.13	*66.96*	*154.86*	10.16	*162.15*	9.39	77.65	3.18	77.77	6.13

Precision and accuracy were assessed by analysis of quadruplicate QC samples on the same day (intra-day) or three consecutive validation days (inter-day). Mouse plasma samples were hydrolyzed by enzyme cocktails following the general procedure and then passed through SPE. Samples were analyzed by UHPLC-QE+/MS. Mouse plasma samples were collected from the control group in MGO-treated mouse study.

Table S6. Concentration of fructosyl-lysine and nine AGEs in hydrolyzed enzyme and plasma matrix solution (nM)

	MG-H1	Lactosyl-lysine	CEL	CEA	MOLD	CML	G-H1	glucosepane	Fructosyl-lysine	3DG-H
Enzymes (nM)	1512.55 ± 18.89	9.67 ± 0.27	108.87 ± 0.31	583.03 ± 30.85	2.70 ± 1.43	512.81 ± 10.44	2.09 ± 1.13	257.51 ± 16.63	10864.69 ± 552.17	78.21 ± 0.43
Plasma matrix (nM)	496.95 ± 90.91	46.90 ± 2.03	56.31 ± 2.27	540.77 ± 123.13	6.82 ± 1.52	652.53 ± 46.92	102.53 ± 2.46	175.63 ± 12.21	464186.45 ± 26855.70	977.19 ± 166.35

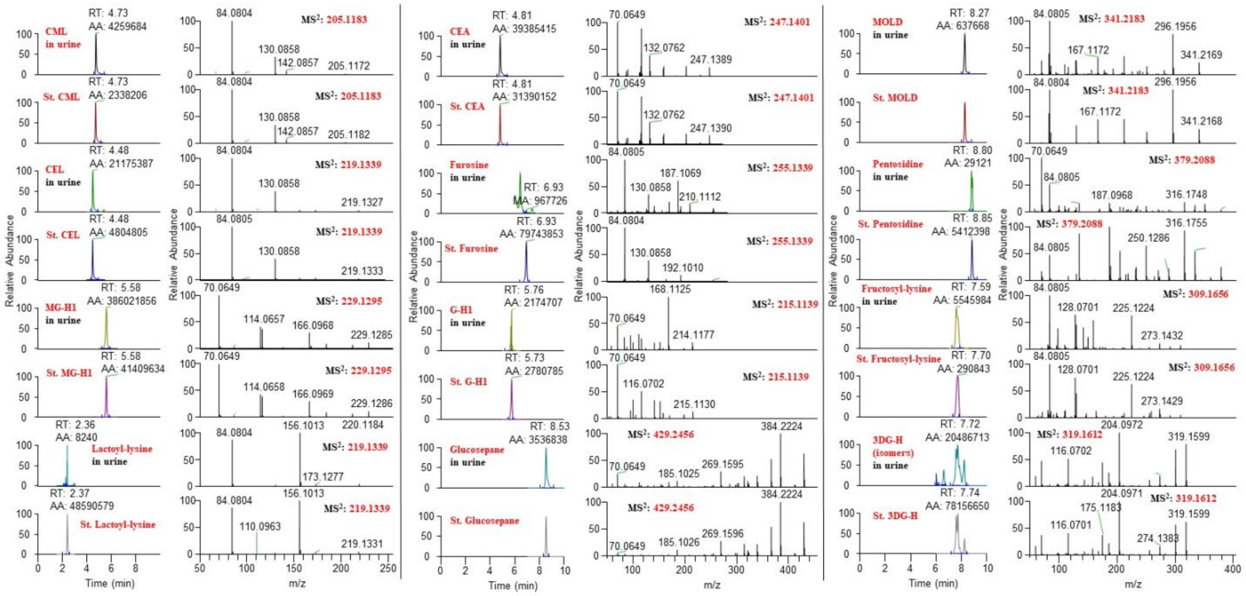

Figure S1. Fructosyl-lysine and eleven AGEs from mouse urine. MS^2 chromatograms and MS^2 spectra of CML, CEL, MG-H1, lactoyl-lysine, CEA, furosine, G-H1, glucosepane, MOLD, pentosidine, fructosyl-lysine, and 3DG-H, in mouse urine, as well as their according standards, obtained by a positive QE+/ESI/MS interface over an Intrada HILIC column. 500 µL urine was passed through a Gilson GX-274 ASPEC with Strata-X-C cartridge. The organic phases were dried. The residue was reconstituted into 50 µL of 50% MeOH with 0.1 N HCl for LC-MS analysis.

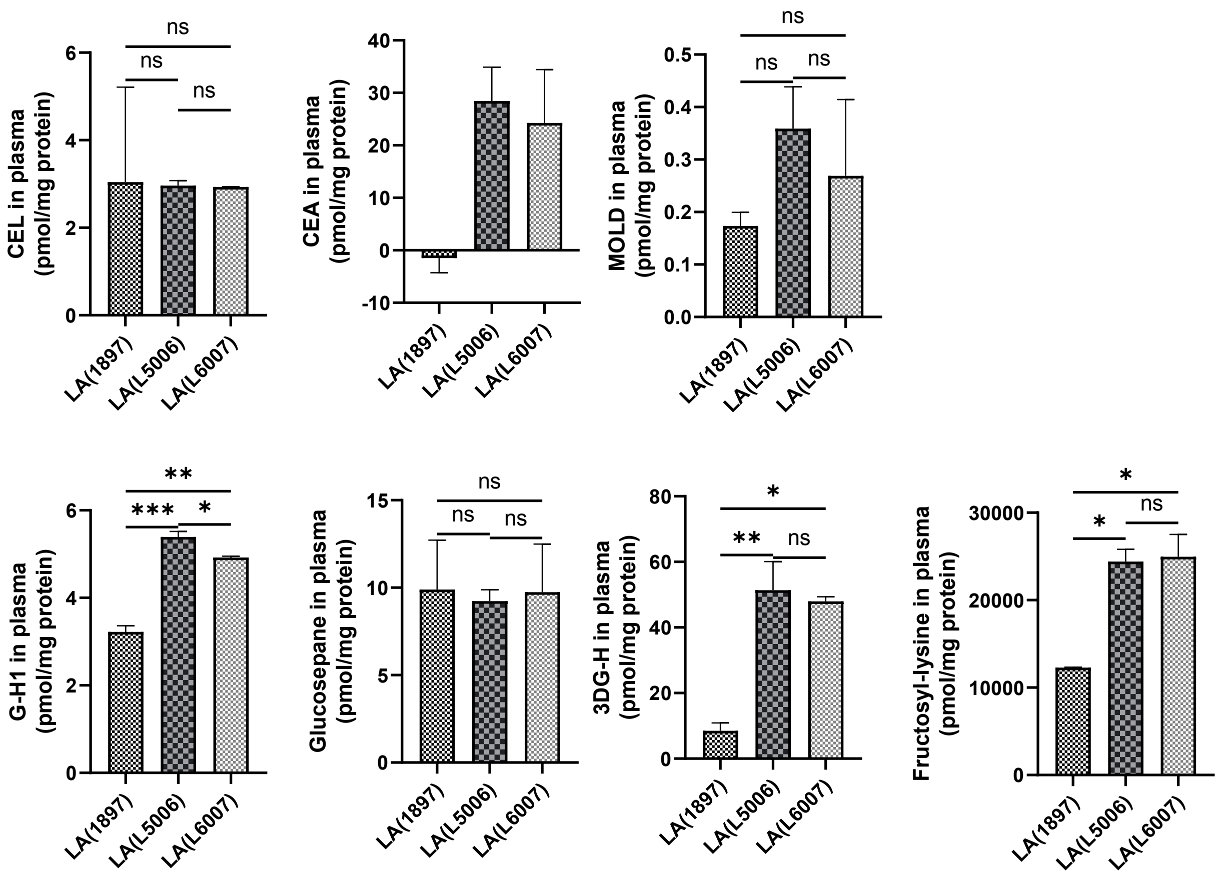

Figure S2. Hydrolytic capacity of leucine aminopeptidase from different sources for AGEs in plasma samples. Fructosyl-lysine and six AGEs in mouse plasma after hydrolysis by different sources of leucine aminopeptidase were determined by a positive QE+/ESI/MS interface over an Intrada HILIC column. Mouse plasma was collected form the control mice in MGO-treated mouse study. Data were expressed as Mean ± SD. Statistical significance ($p \leq 0.05$) was assessed with one-way ANOVA with Tukey's multiple comparisons test using GraphPad Prism 9.4.1.

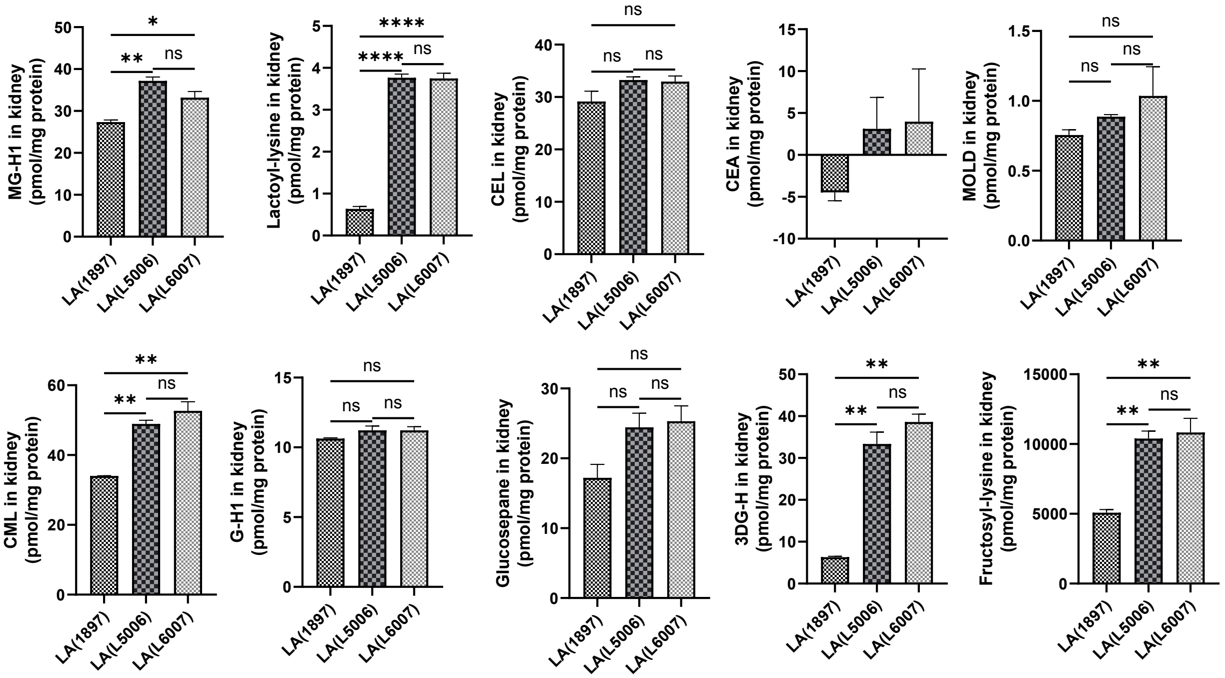

Figure S3. Hydrolytic capacity of leucine aminopeptidase from different sources for AGEs in kidney sample. Fructosyl-lysine and nine AGEs in mouse kidney after hydrolysis by different sources of leucine aminopeptidase were determined by a positive QE+/ESI/MS interface over an Intrada HILIC column. Mouse kidney was collected form the control mice in MGO-treated mouse study. Data were expressed as Mean ± SD. Statistical significance ($p \leq 0.05$) was assessed with one-way ANOVA with Tukey's multiple comparisons test using GraphPad Prism 9.4.1.

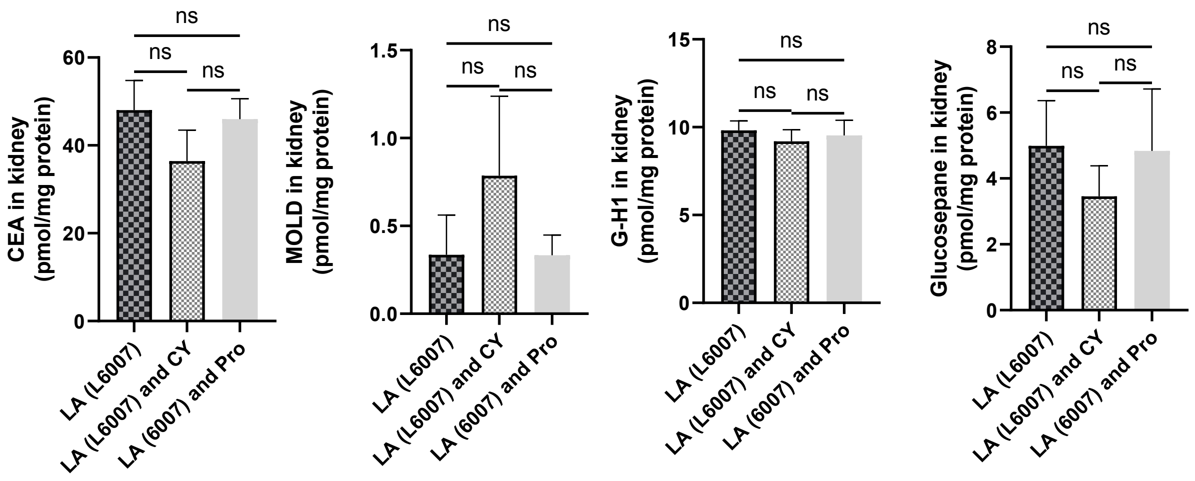

Figure S4. Optimization for enzyme cocktails. Hydrolytic capacity of combinations of leucine aminopeptidase with other enzymes was evaluated. Levels of four AGEs in mouse kidney were determined by a positive QE+/ESI/MS interface over an Intrada HILIC column, following the general procedure for enzymatic hydrolysis. Mouse kidneys were collected form the control mice in MGO-treated mouse study. Data were expressed as Mean ± SD. Statistical significance ($p \leq 0.05$) was assessed with one-way ANOVA with Tukey's multiple comparisons test using GraphPad Prism 9.4.1.

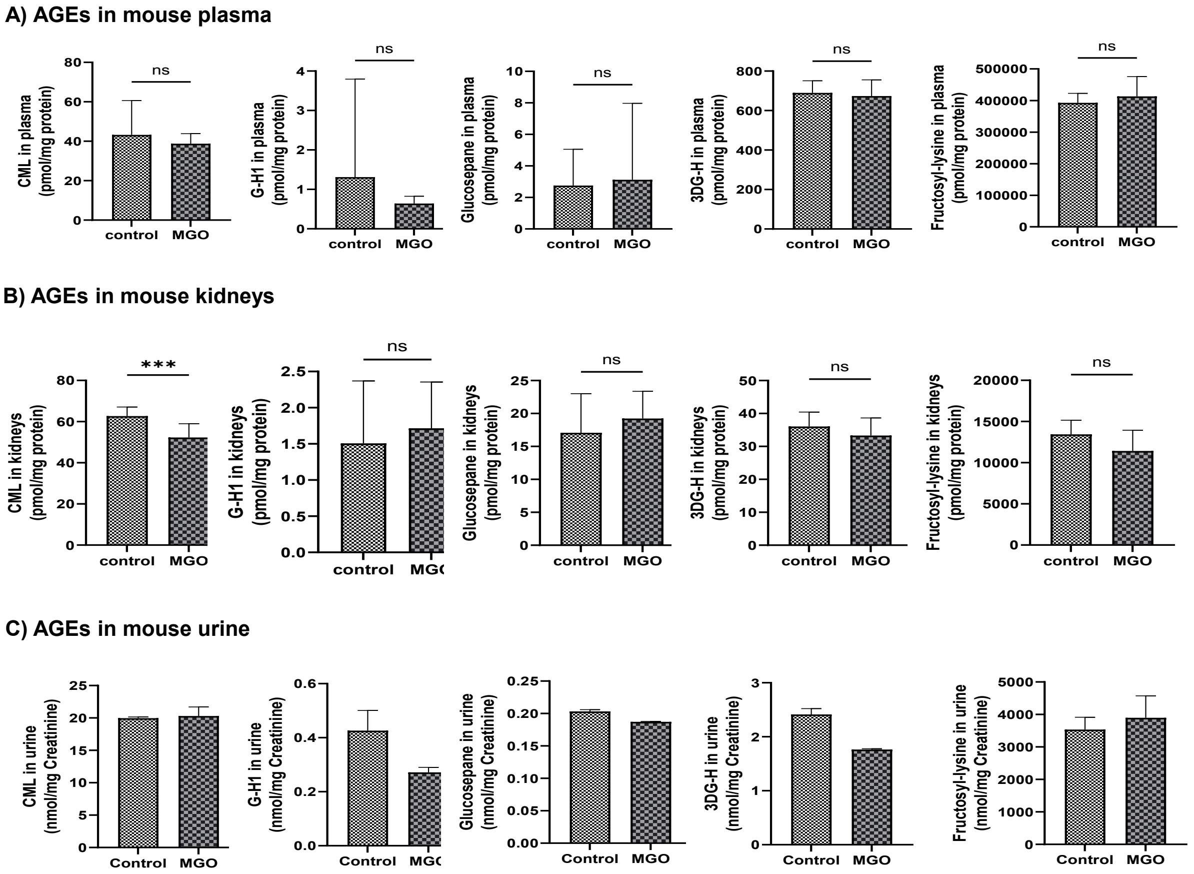

Figure S5. Determination of major AGEs in MGO-treated mice by the optimized method. Levels of fructosyl-lysine and four non-MGO-derived AGEs in mouse plasma (A), kidneys (B), and urine (C) after MGO administration were quantified by a positive QE+/ESI/MS interface over an Intrada HILIC column. CD-1 male mice were administered water or 0.12% MGO in water. Mouse plasma and kidneys were collected after six weeks of treatment. 24-h mouse urine was collected after six weeks of treatment. Data were expressed as Mean ± SD. Statistical significance ($p \leq 0.05$) was assessed with two-tailed non-paired *t*-tests using GraphPad Prism 9.4.1.

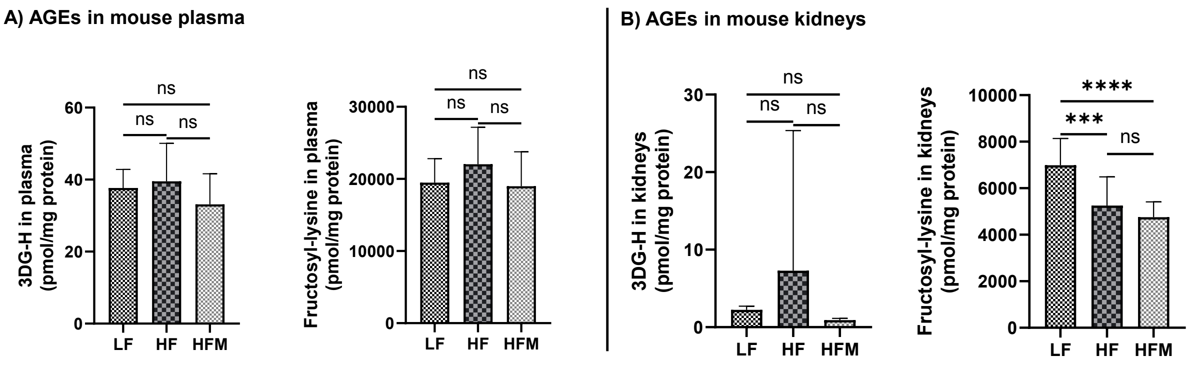

Figure S6. Determination of major AGEs in HF and HF plus MGO-treated mice by the optimized method. Levels of 3DG-H and fructosyl-lysine in mouse plasma (A) and kidneys (B) after LF, HF, and HFM administration were quantified by a positive QE+/ESI/MS interface over an Intrada HILIC column. C57BL/6J male mice were fed with a low fat diet (LF) (10% energy from fat), high fat diet (HF) (45% energy from fat), or high fat diet along with 0.12-0.2% MGO in water (HFM) for 18 wk. At the end of treatments, mice were dissected, and blood samples were harvested. Data were expressed as Mean ± SD. Statistical significance ($p \leq 0.05$) was assessed with one-way ANOVA with Tukey's multiple comparisons test using GraphPad Prism 9.4.1.
